# Comparative genomic and ecological insights into *Salmonella enterica* serovar Kumasi ST2302 from the first Asian clinical isolate

**DOI:** 10.3389/fmicb.2026.1746150

**Published:** 2026-02-27

**Authors:** Shijie Peng, Yan Chen, Taigui Chen, Jun Wang, Peijun Lv, Yanan Wang, Xuebin Xu, Yibin Zhou, Yue Liu

**Affiliations:** 1 Laboratory Department of Panzhihua University, Affiliated Hospital, Panzhihua, China; 2 Department of Microbiology, Panzhihua City Center for Disease Control and Prevention (Panzhihua City Health Supervision Institute), Panzhihua, China; 3 International Joint Research Center of National Animal Immunology, College of Veterinary Medicine, Henan Agricultural University, Zhengzhou, China; 4 Division of Pathogen Testing and Analysis, Shanghai Municipal Center for Disease Control and Prevention, Shanghai, China; 5 Department of Infectious Disease Control, Center for Disease Control and Prevention of Minhang District, Shanghai, China

**Keywords:** cytolethal distending toxin, non-typhoidal *Salmonella*, plant-associated reservoir, *Salmonella enterica* serovar Kumasi, spillover infection genomic sentinel, ST2302, whole-genome sequencing

## Abstract

**Background:**

*Salmonella enterica* subspecies *enterica* serovar Kumasi (*S*. Kumasi) is an exceptionally rare non-typhoidal *Salmonella* (NTS) serovar that has been historically linked to plant- or environment-associated sources and only sporadically isolated from humans. Its genomic characteristics, ecological niche, and pathogenic potential remain poorly defined.

**Methods:**

The clinical isolate (XXB410) underwent antimicrobial susceptibility testing and whole-genome sequencing. Comparative phylogenomic, resistome, virulence, and pan-genome analyses were performed against all 20 publicly available *S*. Kumasi genomes.

**Results:**

We report the first human infection due to *S*. Kumasi in Asia, isolated from a 4-year-old child in China presenting with self-limiting diarrhea. XXB410 was identified as sequence type ST2302 and clustered within a lineage dominated by plant/botanical and non-human sources. The genome was plasmid-free and pansusceptible, carrying only the intrinsic *aac (6’)-Iaa* and *parC* (T57S) polymorphism, with no acquired antimicrobial resistance (AMR) genes. Genomic profiling confirmed the conservation of canonical invasion islands (SPI1/2) and determinants critical for environmental persistence and plant attachment (e.g., *csg* operon), yet revealed an atypical toxin architecture restricted to a divergent *cdtB* homolog without *pltA*/*B* subunits. Pairwise core-genome SNP distances between XXB410 and other ST2302 isolates fell well outside outbreak-level relatedness, supporting sporadic spillover from plant-associated reservoirs rather than sustained human transmission.

**Conclusion:**

These findings expand the clinical and geographic range of *S*. Kumasi and support a model of a plant-adapted lineage characterized by a scarcity of mobile genetic elements (MGEs), an incomplete toxin architecture, and limited clinical expansion. The benign, self-limiting clinical course aligns with the genomic profile. Collectively, we propose *S*. Kumasi as a genomic sentinel to monitor hygiene vulnerabilities in plant-based food supply chains.

## 1 Introduction

Non-typhoidal *Salmonella* (NTS) remains a leading cause of food-borne diarrheal disease worldwide. A landmark global burden analysis estimated approximately 93.8 million episodes of gastroenteritis and 155,000 deaths annually, with the greatest impact in low- and middle-income countries and among children under 5 years of age (Majowicz et al., 2010). Crucially, the globalization of food supply chains has introduced new complexities to pathogen dissemination. A recent global atlas spanning 1900–2023 further quantitatively mapped the evolutionary trajectories of *Salmonella* populations, identifying international food trade and livestock intensification as the primary accelerators of antimicrobial resistance (AMR) dissemination and lineage expansion globally (Wang et al., 2025).

*Salmonella enterica* is highly diverse, comprising more than 2,600 recognized serovars (Grimont and Weill, 2007). While surveillance consistently shows that a limited number of animal-associated serovars, principally S. Enteritidis and S. Typhimurium (including monophasic 4,5,12:i:-), accounts for most human NTS infections globally (Centers for Disease Control and Prevention [CDC], 2025; European Food Safety Authority [EFSA], and European Centre for Disease Prevention and Control [ECDC], 2024; Ferrari et al., 2019; Gal-Mor et al., 2014). This animal-centric focus often obscures a critical blind spot: the rising incidence of outbreaks links to non-animal reservoirs. Epidemiological investigations in North America and Europe have increasingly implicated fresh produce (e.g., leafy greens, onions) and low-moisture foods (e.g., spices and seasonings) as vehicles for widespread salmonellosis (Aiyedun et al., 2021; Centers for Disease Control and Prevention [CDC], 2010; U.S. Food and Drug Administration [FDA], 2025). Notable historical events, such as the multistate *S*. Montevideo outbreak traced to imported red and black pepper (Centers for Disease Control and Prevention [CDC], 2010), underscore the ability of *Salmonella* to persist in desiccated environmental niches and contaminate global food supply chains. Despite this shifting landscape, the specific environmental lineages driving these plant-associated spillovers remain under-surveilled.

To address such surveillance gaps and capture this overlooked diversity, large-scale genomic surveillance efforts have been initiated in China. Through coordinated sequencing and curation of nearly 8,000 isolates, our team established a comprehensive genomic database that integrates human, food, animal, and environmental *Salmonella* data (Wang et al., 2023a; Wang et al., 2023b). This unified resource enables the rapid identification and contextualization of emerging or rare serovars, thereby supporting timely clinical and public-health responses and underscoring the value of sustained genomic monitoring—particularly for environmentally adapted lineages that may act as reservoirs for future spillover, as exemplified by recent findings of diverse antimicrobial resistance genes and plasmids in hospital effluent pathogens (Chen et al., 2025). It was through this systematic surveillance network that the rare isolate described in this study was identified.

The specific lineage identified is *Salmonella enterica* subspecies *enterica* serovar Kumasi (*S*. Kumasi). First described in 1961, this serovar belongs to O-group 30 (formerly Group N), characterized by the antigenic formula 30:z_10_:e,n,z_15_ (Guinée and Kampelmacher, 1961). Unlike broad-host-range serovars, *S*. Kumasi shares ecological traits with other Group N serovars (e.g., *S*. Urbana), historically associated with cold-blooded animals and environmental reservoirs (Kocianová et al., 2010; Rimhanen-Finne et al., 2010). Its environmental niche is strongly substantiated by global surveillance data. Our analysis of the NCBI Pathogen Detection database revealed that majority of *S*. Kumasi genomes were recovered during the 2018 US FDA investigation into contaminated kratom (*Mitragyna speciosa*) products (Schwensohn et al., 2022; U.S. Food and Drug Administration [FDA], 2018). This specific association with a dried botanical commodity strongly supports the hypothesis that *S*. Kumasi occupies a plant-associated ecological niche. In contrast, human clinical isolates are exceptionally rare. Consequently, the isolate (XXB410) reported in this study represents the fourth human infection worldwide and the first clinical report from Asia.

Despite its potential relevance to food safety in plant-based supply chains, *S*. Kumasi remains poorly characterized. The determinants underlying its restricted virulence and sporadic occurrence remain unknown. Here, we describe the genomic features, virulence potential, and ecological context of *S*. Kumasi ST2302. By integrating microbiological identification with whole-genome sequencing (WGS) and comparative analyses, we elucidate the determinants underlying its restricted virulence and propose its utility as a genomic sentinel for plant-associated food safety risks.

## 2 Materials and methods

### 2.1 Case and sample collection

A 4-year-old girl from Sichuan Province, China, presented to the Affiliated Hospital of Panzhihua University on 11 May 2024. A stool specimen was collected for microbiological testing. To identify the etiology, the specimen was subjected to a standard diagnostic workflow, including PCR screening for viral enteric pathogens and selective culture for bacterial pathogens. Clinical information was recorded, and written informed consent was obtained from the patient’s guardians.

### 2.2 Bacterial isolation and identification

The stool specimen was cultured on *Salmonella-Shigella* (SS) agar, xylose lysine deoxycholate (XLD) agar, and Hektoen Enteric (HE) agar plates and incubated at 37 °C overnight. Suspected colonies displaying typically *Salmonella* morphology were picked and subcultured. Species identification was initially performed using Matrix-Assisted Laser Desorption/lonization Time-of-Flight Mass Spectrometry (MALDI-TOF MS)(Bruker Daltonics, Germany) and biochemical profiling via the API 20E system (bioMérieux, France). Serological confirmation was performed by slide agglutination with O and H antisera, and the isolate was assigned to *S.* Kumasi under the Kauffmann-White-Le Minor scheme (antigenic formula 30:z_10_:e,n,z_15_).

### 2.3 Antimicrobial susceptibility testing

Minimum inhibitory concentrations (MICs) were determined by broth microdilution following CLSI guidelines (M100-S33, 2023) (Clinical and Laboratory Standards Institute [CLSI], 2023). Twenty agents from 11 classes were tested: aminoglycosides (amikacin, gentamicin); β-lactam/β-lactamase inhibitor combinations (ampicillin/sulbactam, amoxicillin/clavulanic acid); cephalosporins (cefepime, cefotaxime, cefoxitin, ceftazidime, ceftiofur, cefazolin, cefuroxime); folate pathway inhibitor (trimethoprim-sulfamethoxazole); macrolides (azithromycin); tetracyclines (tetracycline); carbapenems (imipenem); penicillin (ampicillin); phenicols (chloramphenicol); quinolones (nalidixic acid, ciprofloxacin); and polymyxins (polymyxin B). Results were interpreted using CLSI breakpoints. *Escherichia coli* ATCC 25922 served as the quality-control strain.

### 2.4 Whole-genome sequencing and assembly

Genomic DNA was extracted using the QIAamp DNA Mini Kit (Qiagen, Germany). Illumina libraries (2 × 150 bp) were prepared per the manufacturer’s instructions and sequenced on a NovaSeq platform. Reads were quality-filtered with fastp v0.24 (Chen et al., 2018), and assembled using SPAdes v4.0.0 (Bankevich et al., 2012). Assembly metrics were assessed with QUAST v5.3.0 (Gurevich et al., 2013). Annotation was performed with Prokka v1.14.6 (Seemann, 2014).

### 2.5 Genomic analysis

Sequence type (ST) and *in silico* serotyping were determined using the MLST tool (PubMLST) (Jolley et al., 2018) and SeqSero 2 v1.3.2 (Zhang et al., 2019), respectively. To characterize the ecological and pathogenic potential, we screened for antimicrobial resistance (AMR), virulence, and environmental adaptation genes using ABRicate v1.0.1 (Seemann, 2018). Acquired AMR genes were queried against the ResFinder database using strict thresholds (identity ≥ 90%, coverage ≥ 60%). Virulence and adaptation genes were screened against the VFDB database using a minimum identity of 80% and coverage of 60% to capture divergent homologs. Specifically, the amino acid sequence of the divergent *cdtB* allele was extracted and aligned with the reference *S*. Typhi CT18 CdtB (NP_456275.1) using Clustal Omega (Madeira et al., 2024; Sievers et al., 2011), and the alignment was visualized using MView (Brown et al., 1998; Madeira et al., 2024). To ensure structural accuracy, the integrity of *Salmonella* Pathogenicity Islands (SPIs) was further verified using the SPIFinder v2.0 web server^1^ (Roer et al., 2016). Chromosomal mutations and plasmid replicons were identified using PointFinder (Zankari et al., 2017) and PlasmidFinder (Carattoli et al., 2014) (identity ≥ 95%), respectively. Pan-genome analysis was performed using Roary v3.13.0 (Page et al., 2015), with gene presence/absence visualized using the bundled scripts.

For phylogenomic analysis, core-genome SNPs were called using Snippy v4.6.0 (Seemann, 2015). To ensure accurate distance estimation within the lineage, the high-quality draft genome of *S.* Kumasi ST2302 (RefSeq GCA_005902525.1; 44 contigs; N50 = 268,613 bp) was selected as the mapping reference. Putative recombination was detected and masked using Gubbins v3.4 (Croucher et al., 2015). A recombination-corrected maximum-likelihood phylogeny was reconstructed using IQ-TREE 2 under the GTR++G model with 1,000 ultrafast bootstrap replicates (Hoang et al., 2018; Minh et al., 2020).

### 2.6 Ethical statement

The study involving human participants was reviewed and approved by the Ethics Committee of the Affiliated Hospital of Panzhihua University (Approval No. 2025-11-007). This study adhered to the Declaration of Helsinki. Written informed consent was obtained from the patient’s guardians for the publication of this case report and any accompanying data. All personally identifiable information was anonymized.

### 2.7 Data availability

The genome sequence of *S.* Kumasi isolate XXB410 has been deposited in the GenBank under accession number JBRSFH000000000 (BioProject PRJNA1311721; BioSample SAMN50839459).

## 3 Results

### 3.1 Clinical presentation and isolate identification

The patient presented with acute watery diarrhea with visible blood streaks. Routine microbiological screening yielded negative results for common viral agents (Rotavirus and Norovirus) and bacterial pathogens (e.g., *Shigella* spp.). *S*. Kumasi was the sole pathogen recovered from the stool culture, confirmed by biochemical assays and serological test (antigenic formula 30:z_10_:e,n,z_15_). The clinical course, laboratory findings, and management are summarized in Supplementary Table 1.

### 3.2 Genomic features of isolate XXB410

The XXB410 draft genome comprised 4.57 Mb assembled into 51 contigs (N50, 262,886 bp; largest contig, 594,055 bp; GC content, 52.2%). SeqSero2 predicted the serovar as *S*. Kumasi, consistent with traditional serological test. MLST assigned the isolate to sequence type ST2302. To our knowledge, this represents the fourth human infection worldwide and the first reported case from Asia.

### 3.3 Global genomic context and ecological clustering

Analysis of comprehensive 21 genomes dataset identified ST2302 as the dominant lineage (19/21). Epidemiological metadata revealed a strong ecological association: nearly all historical isolates originated from environmental or plant sources, including kratom products sampled by the US FDA, whereas human cases remain exceptional (Supplementary Table 2).

**FIGURE 1:**
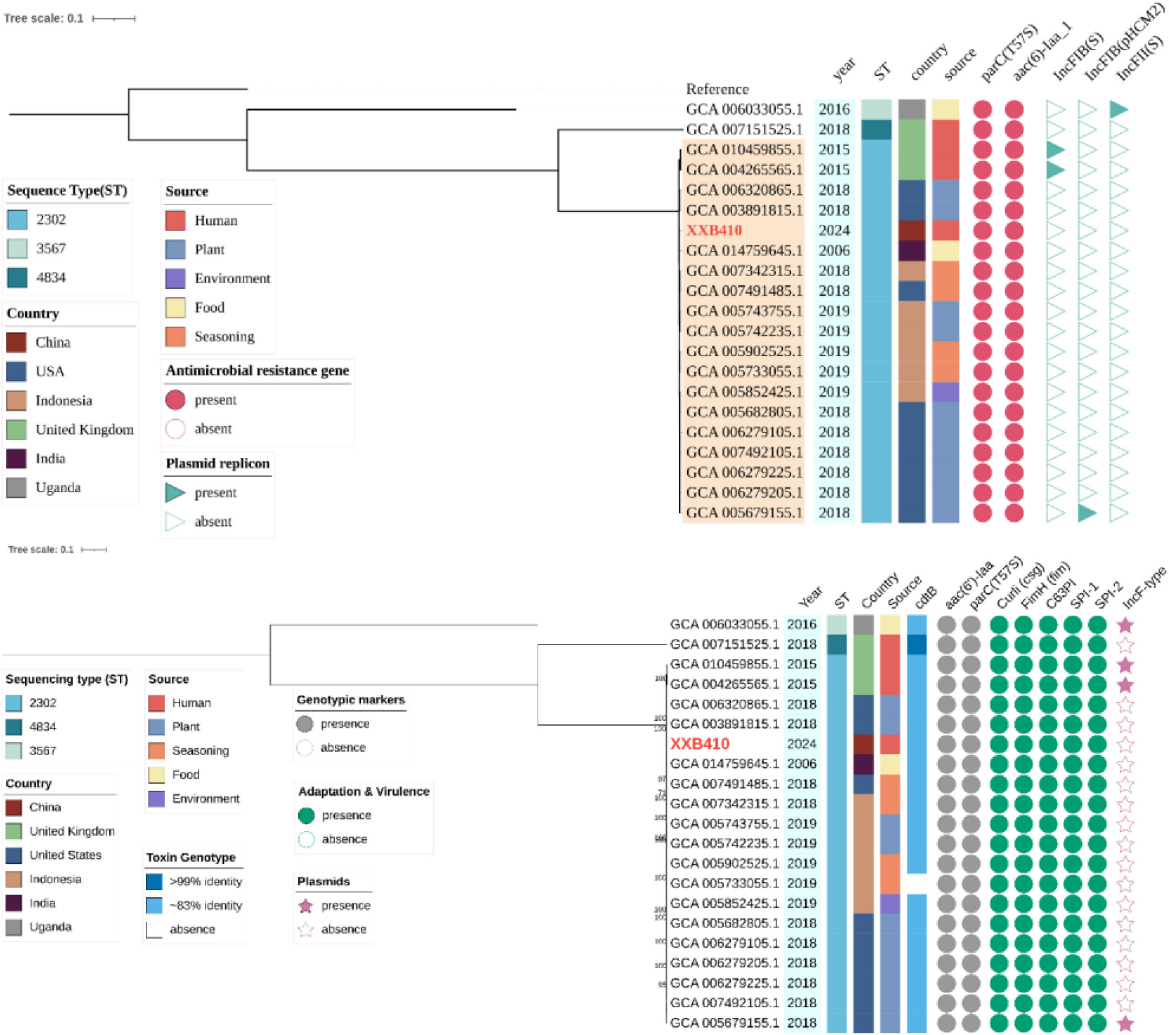
Global phylogenomic context of *S*. Kumasi. Maximum-likelihood phylogeny based on core-genome SNPs, referencing the *S*. Kumasi genome (GCA_005902525.1). The tree was reconstructed using IQ-TREE 2 with 1,000 ultrafast bootstrap replicates. Node labels indicate bootstrap support value > 70%. The scale bar represents nucleotide substitutions per site. Annotations (left to right): metadata, year, sequence type (ST), country, and isolation source; toxin genotype, presence of the *cdtB* gene; genotypic markers, presence of the intrinsic *aac (6’)-Iaa* gene and the *parC* (T57S) polymorphism; adaptation and virulence, presence of plant-adaptation determinants and intact *Salmonella* Pathogenicity Islands; plasmids, presence of IncF-type plasmid replicons.

Phylogenomic analysis placed isolate XXB410 firmly within the environmental ST2302 clade (Figure 1). Pairwise SNP analysis revealed that XXB410 differs from its closest relatives by 115 SNPs. This genetic distance exceeds the threshold typically defined for direct outbreak transmission (Ashton et al., 2015), supporting a model of sporadic spillover from a globally distributed plant-associated reservoir rather than sustained human-to-human transmission.

### 3.4 Environmental adaptation and genomic stability

**FIGURE 2:**
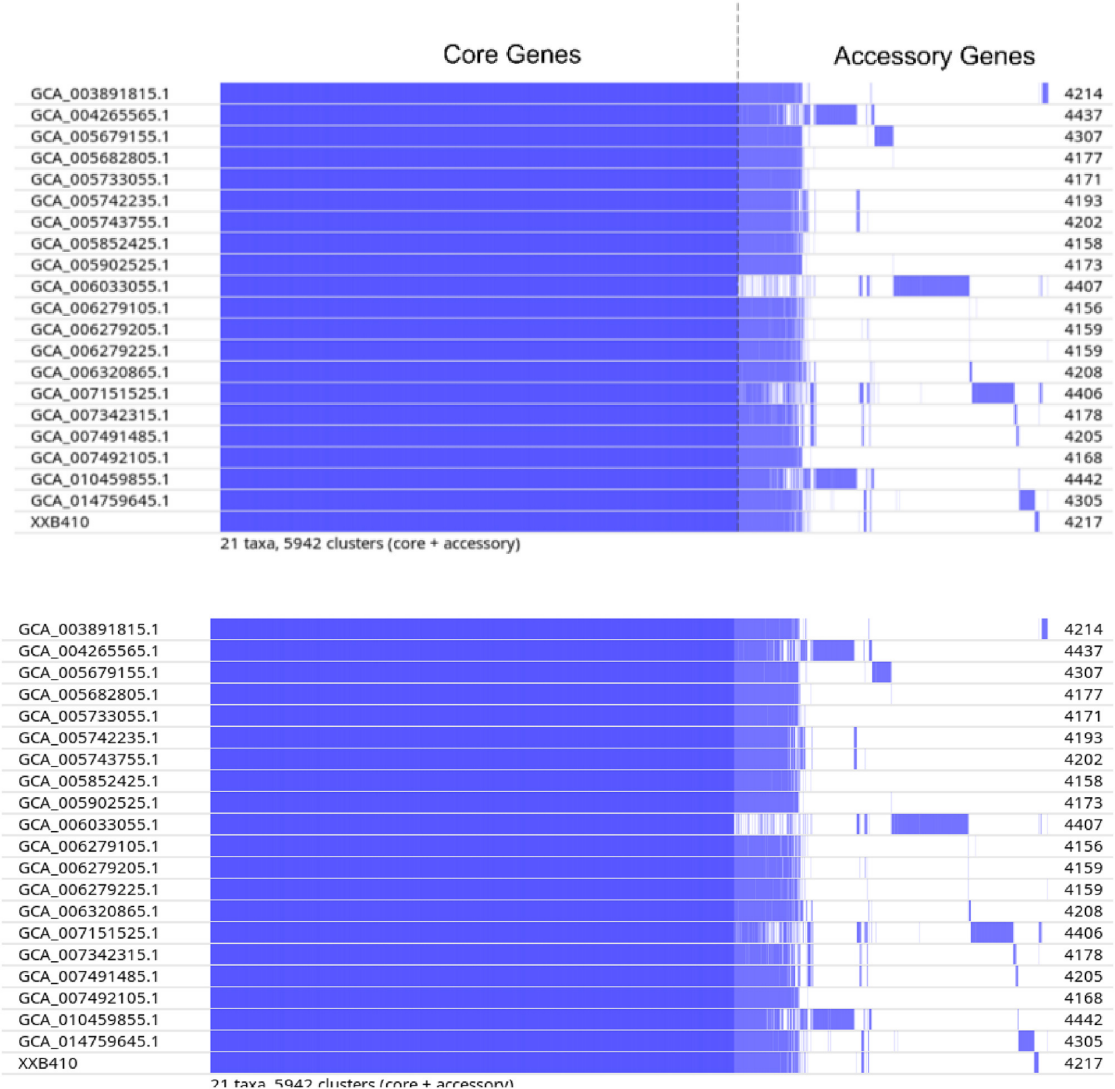
Pan-genome analysis of 21 *S*. Kumasi genomes. The heatmap shows the presence and absence of accessory genes across isolates, aligned to the core-genome phylogeny. The vertical dashed line delineates the boundary between the conserved core genome (left) and the variable accessory genome (right). Gene categories (core, shell, cloud) are indicated by color coding intensity.

Pan-genome analysis (Figure 2) identified a total of 5,942 gene clusters across the 21 genomes dataset. The core genome comprised 3,714 clusters, accounting for 62.5% of the total gene pool, while the accessory genome consisted of 2,228 clusters. Within the conserved core genome, screening for environmental adaptation markers revealed the universal presence of the *csg* operon (encoding curli fimbriae) and the *fim* operon (encoding Type 1 fimbriae) in all isolates, with nucleotide identities exceeding 99% (Supplementary Table 2). Additionally, the C63PI pathogenicity island (typically encoding the *sit* iron-uptake system) was detected in all genomes via SPIFinder analysis (Supplementary Table 3).

Regarding genomic plasticity, plasmid replicons were absent from the clinical isolate XXB410 and were detected in only a minority of the global genomes (IncF-type) (Figure 1). Correspondingly, no acquired antimicrobial resistance (AMR) genes were identified in the ST2302 lineage. The chromosomal genotype was restricted to the intrinsic *aac (6’)-Iaa* gene and *parC* (T57S) polymorphism. This genomic profile corresponded with the phenotypic susceptibility of XXB410 to all 20 antimicrobial agents tested (Supplementary Table 4).

### 3.5 Virulence architecture

Virulence profiling identified a conserved set of invasion determinants but an incomplete toxin locus. Analysis using SPIFinder confirmed the presence of *Salmonella* Pathogenicity Islands 1 (SPI-1) and 2 (SPI-2) across all 21 genomes, characterized by high nucleotide identities ( > 97%) and canonical insertion sites (Figure 1 and Supplementary Table 3).

**FIGURE 3:**
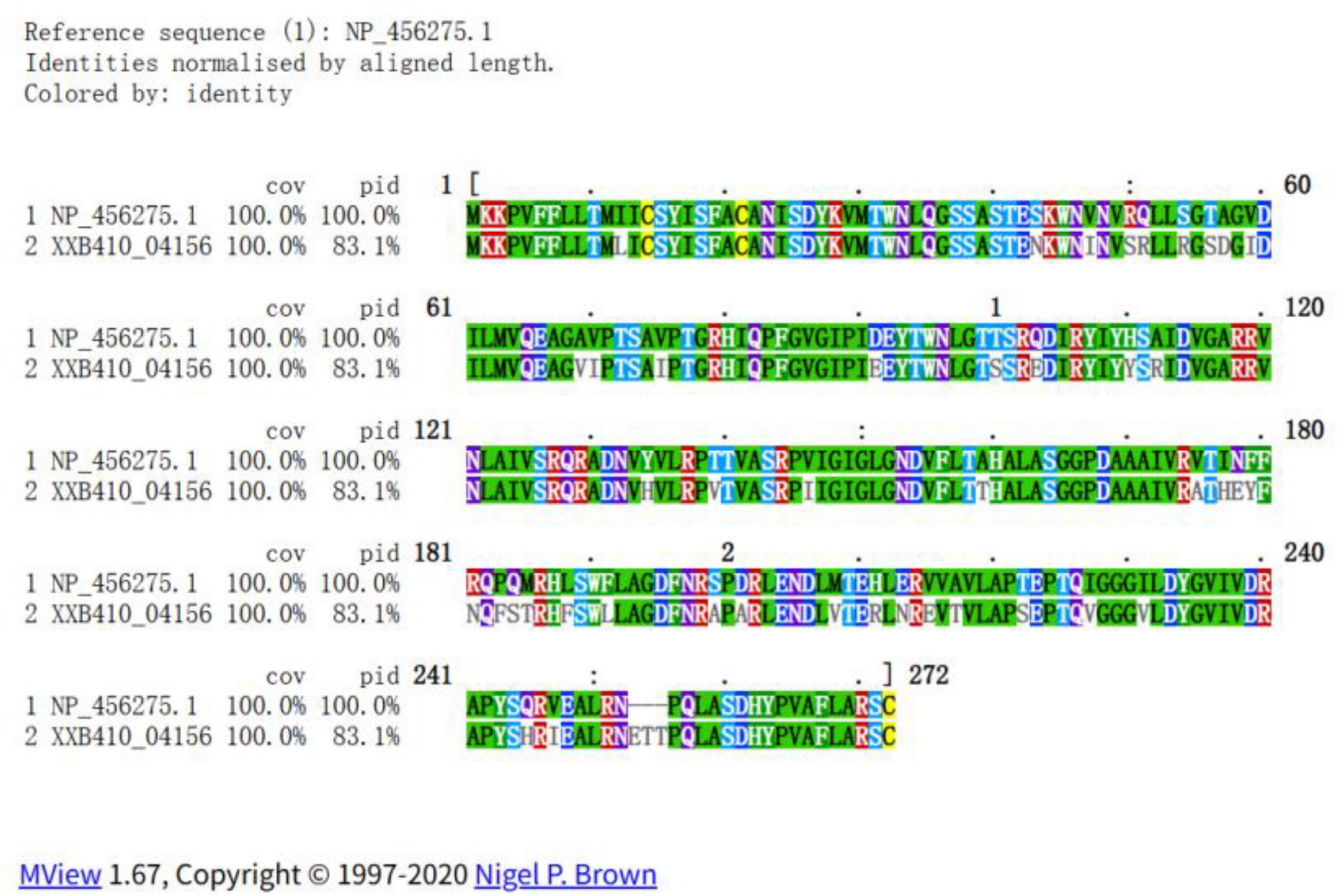
Amino acid sequence alignment of CdtB. Pairwise alignment of the CdtB protein from *S*. Kumasi (XXB410) and the reference *S*. Typhi CT18 (Accession: NP_456275.1). Key metrics: 100% Coverage (cov) indicates the *S*. Kumasi (XXB410) is a full-length protein with no truncations. 83.1% Identity (pid) highlights the significant sequence divergence from the canonical typhoid toxin.

In contrast, the typhoid toxin locus exhibited lineage-specific variation regarding the *cdtB* subunit, yet the holotoxin structure remained incomplete across the entire dataset (Supplementary Table 2). While a cdtB homolog was detected in all genomes, the essential *pltA* and *pltB* delivery subunits were absent in all 21 isolates. Specifically, the ST2302 lineage (including XXB410) carried a divergent *cdtB* allele (∼83% amino acid identity to *S*. Typhi). Sequence alignment revealed that despite this divergence, the protein retains conserved catalytic residues essential for DNase activity (Figure 3). However, the universal absence of the delivery complex suggests that holotoxin assembly is impaired in this serovar. Notably, a canonical *cdtB* allele ( > 99% identity) was identified only in the single ST4834 isolate, consistent with the broader lineage, it also lacked the accompanying *pltA* and *pltB* subunits.

## 4 Discussion

This study broadens the clinical and geographic footprint of *S.* Kumasi by characterizing the first human case reported in Asia. By integrating the clinical genome with global public datasets, we propose that *S*. Kumasi represents a specialized plant-adapted lineage rather than a typical broad-host-range serovar. This ecological positioning is strongly substantiated by the origin of the global dataset: a significant proportion of the available genomes were isolated during the 2018 US FDA investigation into contaminated kratom products (Schwensohn et al., 2022; U.S. Food and Drug Administration [FDA], 2018). The recovery of *S*. Kumasi from these dried botanical supplements not only clarifies the epidemiological background of these archival strains but also, given its rarity in extensive global livestock surveillance, strongly points toward a predominantly plant-associated or environmental ecological niche. To persist in such desiccated, nutrient-limited environments, *Salmonella* requires specific genomic mechanisms, which are reflected in the *S*. Kumasi genome.

The genomic machinery of *S*. Kumasi strongly corroborates its environmental adaptability. Our analysis revealed the universal conservation of the *csg* operon (curli fimbriae) and the *fim* operon (Type 1 fimbriae) across all genomes. Functionally, the FimH adhesin specifically binds mannose residues on plant cell walls, while the *csg* operon encodes curli fimbriae—the primary structural component of the “rdar” (red, dry, and rough) biofilm morphotype (Barak et al., 2005; Berger et al., 2010; White et al., 2006; Zogaj et al., 2001). This phenotype is critical for protecting *Salmonella* against desiccation stress and UV radiation (White et al., 2006). Furthermore, the detection of the C63PI island suggests an enhanced capacity to sequester iron via the *sit* system in the metal depleted phyllosphere (Brandl, 2006). Collectively, this specific genomic toolkit aligns with the archetypal features of plant-associated *Salmonella*, supporting the classification of *S*. Kumasi as a lineage specialized for survival in the phyllosphere and dried botanical commodities.

The distinct environmental niche also provides a plausible explanation for the lineage’s genomic stability and lack of antimicrobial resistance and plasmids. Unlike high-burden serovars that evolve under constant clinical antibiotic pressure, *S*. Kumasi displays a pansusceptible phenotype and a scarcity of plasmids. While genomic screening identified an intrinsic *aac (6’)-Iaa* gene and a *parC* (T57S) polymorphism, crucially, neither of these features conferred phenotypic resistance. The *parC* (T57S) substitution is widely recognized as a natural phylogenetic marker rather than a resistance-conferring mutation (Chang et al., 2021; Vázquez et al., 2022), and the *aac (6’)-Iaa* gene is typically silent in *Salmonella* unless upregulated by specific mutations (Magnet et al., 1999; Tang et al., 2022). This profile reflects an evolutionary history in “low-pressure” non-clinical environments. However, despite this adaptation, *S*. Kumasi appears to retain a “cross-kingdom lifestyle” capacity characteristic of the genus (Schikora et al., 2012). The conservation of intact SPI-1and SPI-2 invasion systems indicates that the lineage retains the genomic potential to colonize mammalian hosts upon ingestion (Sia et al., 2025), providing a biological basis for the diarrheal symptoms observed in our case.

Regarding pathogenicity, the isolate presents an “atypical” virulence profile. While canonical invasion determinants (SPI-1 and SPI-2) are retained, the typhoid toxin locus is incomplete. Crucially, sequence analysis reveals that while the divergent CdtB homolog retains conserved catalytic residues essential for DNase activity, the absence of the delivery complex likely compromises the toxin’s entry into host cells (Song et al., 2013). This pattern of gene loss resembles host-adaptation trajectories described in other lineages and aligns with the self-limiting nature of the infection (Langridge et al., 2015; Zhou et al., 2023). Clinically, although incidental detection via foodborne transport is a theoretical possibility, the definitive exclusion of other common viral and bacterial pathogens establishes *S*. Kumasi as the sole recovered agent, strongly supporting its role as the primary cause.

We acknowledge certain limitations in this study. First, our conclusions regarding the “atypical” virulence potential and environmental adaptation are based on high-resolution genomic data. Future functional characterization using *in vitro* or *in vivo* models is required to definitively confirm the biological activity of the divergent *cdtB* and the biofilm-forming capacity. Second, establishing definitive causality in a single case report presents inherent challenges. While we rigorously excluded common viral and bacterial co-pathogens, the theoretical possibility of incidental detection cannot be entirely eliminated without broader epidemiological data.

From a public health perspective, we propose that *S*. Kumasi serves as a valuable “genomic sentinel”. While the serovar itself may pose a lower immediate clinical risk than high-burden serovars, its detection signals hygiene vulnerabilities in non-animal supply chains, particularly fresh produce and herbal supplements, which are increasingly recognized as vehicles for transboundary salmonellosis (Aiyedun et al., 2021; Schwensohn et al., 2022). Monitoring such sentinel lineages allows for the early detection of contamination routes that might be missed by traditional meat-focused surveillance. Finally, we emphasize the critical importance of international collaboration. By contributing this first Asian clinical genome to public repositories, we underscore the necessity of global data transparency and an One Health approach to track the cross-border movement of environmental pathogens in our interconnected food systems.

## 5 Conclusion

*S*. Kumasi appears to be a plant-associated lineage with an incomplete typhoid-toxin architecture and limited clinical expansion. These features argue for targeted genomic surveillance of plant-linked supply chains to detect and prevent sporadic spillover.

## Data Availability

The datasets presented in this study can be found in online repositories. The names of the repository/repositories and accession number(s) can be found in the article/Supplementary material.

## Footnotes

1
https://cge.food.dtu.dk/services/SPIFinder/
